# Fascial Dehiscence and Incisional Hernia Prediction Models: A Systematic Review and Meta-analysis

**DOI:** 10.1007/s00268-022-06715-6

**Published:** 2022-09-14

**Authors:** Amarit Tansawet, Pawin Numthavaj, Thawin Techapongsatorn, Suphakarn Techapongsatorn, John Attia, Gareth McKay, Ammarin Thakkinstian

**Affiliations:** 1 Department of Clinical Epidemiology and Biostatistics, Faculty of Medicine Ramathibodi Hospital, Mahidol University, Rama VI Road, Bangkok, Ratchathewi, 10400 Thailand; 2 Department of Surgery, Faculty of Medicine Vajira Hospital, Navamindradhiraj University, Bangkok, Thailand; 3 Faculty of Medicine, King Chulalongkorn Memorial Hospital, Chulalongkorn University, Bangkok, Thailand; 4 Centre for Clinical Epidemiology and Biostatistics, School of Medicine and Public Health, Hunter Medical Research Institute, University of Newcastle, New Lambton, NSW Australia; 5 Centre for Public Health, School of Medicine, Dentistry, and Biomedical Sciences, Queen’s University Belfast, Belfast, Northern Ireland, UK

## Abstract

**Background:**

Fascial dehiscence (FD) and incisional hernia (IH) pose considerable risks to patients who undergo abdominal surgery, and many preventive strategies have been applied to reduce this risk. An accurate predictive model could aid identification of high-risk patients, who could be targeted for particular care. This study aims to systematically review existing FD and IH prediction models.

**Methods:**

Prediction models were identified using pre-specified search terms on SCOPUS, PubMed, and Web of Science. Eligible studies included those conducted in adult patients who underwent any kind of abdominal surgery, and reported model performance. Data from the eligible studies were extracted, and the risk of bias (RoB) was assessed using the PROBAST tool. Pooling of C-statistics was performed using a random-effect meta-analysis. [Registration: PROSPERO (CRD42021282463)].

**Results:**

Twelve studies were eligible for review; five were FD prediction model studies. Most included studies had high RoB, especially in the analysis domain. The C-statistics of the FD and IH prediction models ranged from 0.69 to 0.92, but most have yet to be externally validated. Pooled C-statistics (95% CI) were 0.80 (0.74, 0.86) and 0.81 (0.75, 0.86) for the FD (external-validation) and IH prediction model, respectively. Some predictive factors such as body mass index, smoking, emergency operation, and surgical site infection were associated with FD or IH occurrence and were included in multiple models.

**Conclusions:**

Several models have been developed as an aid for FD and IH prediction, mostly with modest performance and lacking independent validation. New models for specific patient groups may offer clinical utility.

**Supplementary Information:**

The online version contains supplementary material available at 10.1007/s00268-022-06715-6.

## Introduction

Abdominal surgery is one of the most common operations worldwide. Although surgical techniques and perioperative care have improved dramatically, wound complications including fascial dehiscence (FD) and incisional hernia (IH) still occur. FD occurs in about 0.24–5.8% of post-laparotomy patients and carries an increased risk of mortality (approximating 25%) [1]. The incidence of IH ranges from 5 to 20%, increasing up to 30% in high-risk patients [2] and after FD occurrence [3]. As such, a significant amount of healthcare resources could be saved if the incidence of FD and IH occurrence could be reduced [4].

Perioperative risk optimization (such as prevention of wound infection [1, 5, 6], preoperative smoking cessation [7, 8], and bodyweight reduction [6]) is essential for FD and IH prevention. In addition, mesh techniques have recently been improved for FD and IH prophylaxis [9–11]. Hence, the risk associated with these adverse events can be reduced through an intensive prevention strategy but targeting these enhanced methods to those at highest risk would be more cost effective than using them routinely for all patients. An accurate risk prediction model for FD and IH would help identify patients at greater risk of FD and IH occurrence and therefore provide more selective allocation of prevention interventions.

This systematic review was therefore conducted to identify FD and IH risk prediction models available in the literature. Evidence was summarized in terms of risk factors, statistical models used, model performance, and associated risk of bias. Model performance was described by study phases including derivation, internal-validation, and external-validation.

## Material and methods

A review protocol was developed following the PRISMA2020 guideline [12] (Online Appendix 1) and registered in PROSPERO (CRD42021282463).

### Study identification and selection

SCOPUS, Medline (via PubMed), and Web of Science databases were used for study identification from inception to September 28th, 2021. Search terms were constructed using keywords as follows: incisional hernia, dehiscence, prediction model, receiver operating characteristic (ROC) curve, concordance statistic (C-statistic), sensitivity, specificity, derivation, and validation. Synonyms of these terms were also considered (Online Resource Table S1). Studies published in any language were eligible if they met the following criteria: developed or validated a risk prediction model of FD or IH in adult patients who underwent abdominal surgery, included more than one risk factor in the risk prediction model, and reported their model’s performance (i.e., C-statistic, sensitivity, specificity, predictive values, and observed/expected (O/E) outcome ratio). Studies were excluded if their aim was to examine FD or IH prediction in open abdomen, ventral hernia treatment, and parastomal hernia. Two reviewers (ATa and TT) independently selected the eligible studies. Disagreements were resolved by the third reviewer (ST).

### Data extraction and risk of bias assessment

Study level data were extracted by two reviewers (ATa and TT) including study design (i.e., cohort or case–control), the number of patients and events of interests, and patients’ demographics and characteristics. In addition, the study phase (i.e., derivation or validation), type and number of risk factor, type of statistical model, and model selection were also extracted.

Furthermore, model performance reported as C-statistic along with 95% confidence interval (CI), sensitivity, specificity, and predictive values were extracted. If the 95% CI of a C-statistic was not reported, it was calculated using the equation proposed by Hanley and McNeil [13]. The calibration performance (assesses how close the predicted and the actual values are, measuring by the Hosmer–Lemeshow goodness-of-fit chi-square test or the O/E ratio, or both), was retrieved if data were available.

The individual study risk of bias (RoB) was assessed using the PROBAST tool [14]. This tool consists of four components including participants, predictors, outcome, and analysis domains. There are two to nine signal questions for each domain with a total of 20 questions. Each study was finally classified as high RoB if at least one domain was rated as high risk, low RoB if all domains were rated as low risk, and unclear if the result of assessment was unclear. Two reviewers (ATa and ST) independently performed RoB assessment, and any disagreement was resolved by consensus.

### Meta-analysis

C-statistics, along with standard errors (SE), were described. The SEs were estimated from reported 95% CIs or the equation proposed by Hanley and McNeil [13]. These C-statistics were then pooled across studies where data were available using a random-effect model if heterogeneity was present. Heterogeneity was assessed by the I^2^-statistic; I^2^ > 25% indicated the presence of heterogeneity. All analyses were stratified by type of prediction models (FD and IH) and study phases and displayed with Forest plots where data were available. Meta-regression, sensitivity analysis, and publication bias assessment were planned but could not be performed because of the limited number of included studies. STATA version 17 (StataCorp, Texas, USA) was used for all analyses. Certainty of the evidence was rated according to the Grade of Recommendation, Assessment, Development, and Evaluation (GRADE) guideline [15, 16].

## Results

**Fig. 1:**
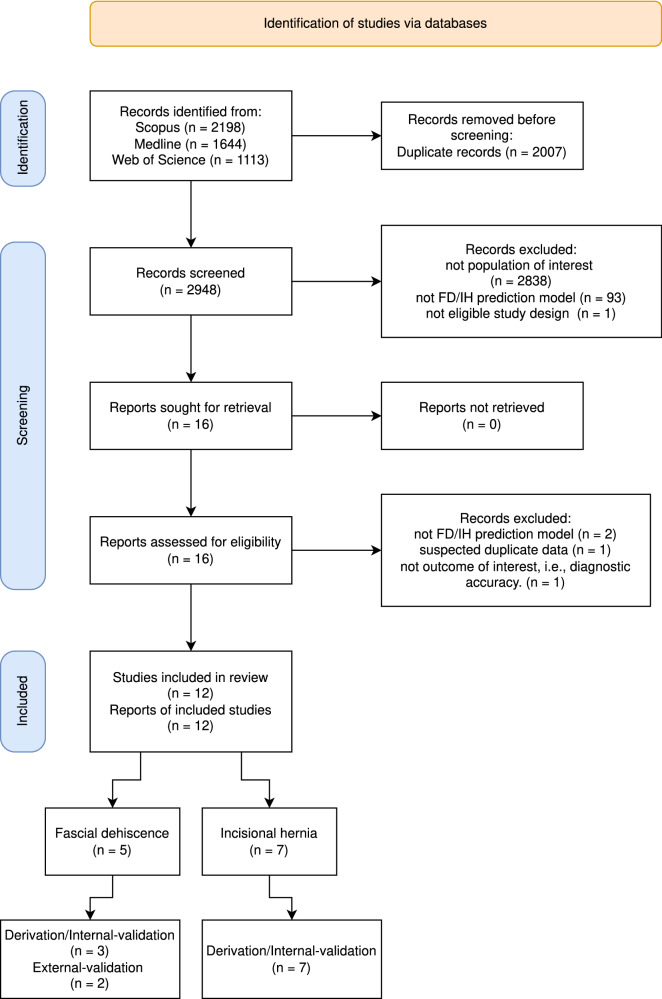
PRISMA flow of study selection

Twelve out of 2, 948 studies [17–28], comprising 209, 104 patients, were eligible including 5 FD [17, 18, 20, 21, 28] (3 derivation/internal-validation and 2 external-validation) and 7 IH [19, 22–27] (all derivation/internal-validation) studies (Fig. 1). No study that appeared to meet inclusion criteria was later excluded. The mean age ranged from 45.3 to 66.8 years; percentage male was 26.6–93.9% (Table 1). Only 4/12 studies reported body mass index (BMI) which ranged from 28.2 to 56.8 kg/m^2^. Ten out of 12 eligible studies were cohort designs with follow up time from 6 to 57 months.

**Table 1:** Characteristics of the included studies

Study	Population	Incision (approach)	Data source	Study design	Phase	Patients (n)	Events (n)	Age, year (mean)	Sex, male (%)	BMI, kg/m^2^ (mean)	Follow-up, month
FD prediction model											
*VAMC model*											
Webster, 2003	Mixed abdominal surgery	Open	NSQIP	Cohort	Derivation	17, 044	587	n/a	n/a	n/a	n/a
					Internal-validation	17, 763	562	n/a	n/a	n/a	n/a
Kenig, 2014	Mixed abdominal surgery	Open	Medical records	Case–control	External-validation	224	56	66.8	58.9	n/a	n/a
*Rotterdam model*											
Ramshorst, 2010	Mixed abdominal surgery	Open	Medical records	Case–control	Derivation	1, 452	363	59	60.3	n/a	n/a
					Internal-validation	686	19	58.2	60	n/a	n/a
Días, 2014	Mixed abdominal surgery	Open	Medical records	Cohort	External-validation	176	15	n/a	60.2	n/a	n/a
Kenig, 2014	Mixed abdominal surgery	Open	Medical records	Case–control	External-validation	224	56	66.8	58.9	n/a	n/a
*Machine learning model*											
Cole, 2021	Mixed abdominal surgery	Open	NSQIP	Cohort	Derivation	69, 969	1, 332	61	42.1	28.3	n/a
					Internal-validation	23, 055	390	61	42.3	28.6	n/a
IH prediction model											
Veljkovic, 2010	Mixed abdominal surgery	Open	Prospective data	Cohort	Derivation	603	81	59	53.7	n/a	> 6
Goodenough, 2015 (HERNIAscore)	Mixed abdominal surgery	Open/Lap	Prospective data	Cohort	Derivation	428	70	60.7	93.9	28.2	41
					Internal-validation	197	23	n/a	n/a	n/a	41
Basta, 2016	Bariatric	Open/Lap	Medical records	Cohort	Derivation	2, 161	52	45.3	26.6	56.8	28.3
Fischer, 2016	Mixed abdominal surgery	Open	Medical records	Cohort	Derivation	12, 373	436	55.9	33.4	n/a	32.2 ± 26.6
Lanni, 2016^a^	Colectomy	Open/Lap	Medical records	Cohort	Derivation	30, 865	1, 698	n/a	n/a	n/a	30
Tecce, 2017	Hysterectomy	Open	Medical records	Cohort	Derivation	2, 145	76	52.6	n/a	n/a	n/a
Basta, 2019 (Penn risk calculator)	Mixed abdominal surgery	Open/Lap	Medical records	Cohort	Mixed derivation and internal-validation	29, 739 19, 799/9, 940^b^	1, 127	52.6	36.6	30.1	57.9

*BMI*: Body mass index, *FD*: fascial dehiscence, *IH*: incisional hernia, Lap Laparoscopic surgery, *n*/*a*: Not available, *NSQIP*: National surgical quality improvement program, *VAMC*: Veteran affairs medical centre

^a^No full-text available

^b^Derivation/internal-validation

RoBs were assessed for all studies using the PROBAST, except one [25] due to a full-text unavailability. Most included studies were judged as high RoB, particularly in the domain of participants and data analysis, see Online Resource Table S2.

### FD Prediction

For the 5 FD prediction models, 3 and 2 studies were derivation plus internal-validation and external-validation phases, respectively. Three risk prediction models were derived by groups from the Veteran Affairs Medical Centre (VAMC) [17], Rotterdam [18], and Virginia [28] using the data of 17, 044, 1, 452, and 69, 969 patients; these models were also internally validated in 17, 763, 686, and 23, 055 patients, respectively (Table 1).

**Fig. 2:**
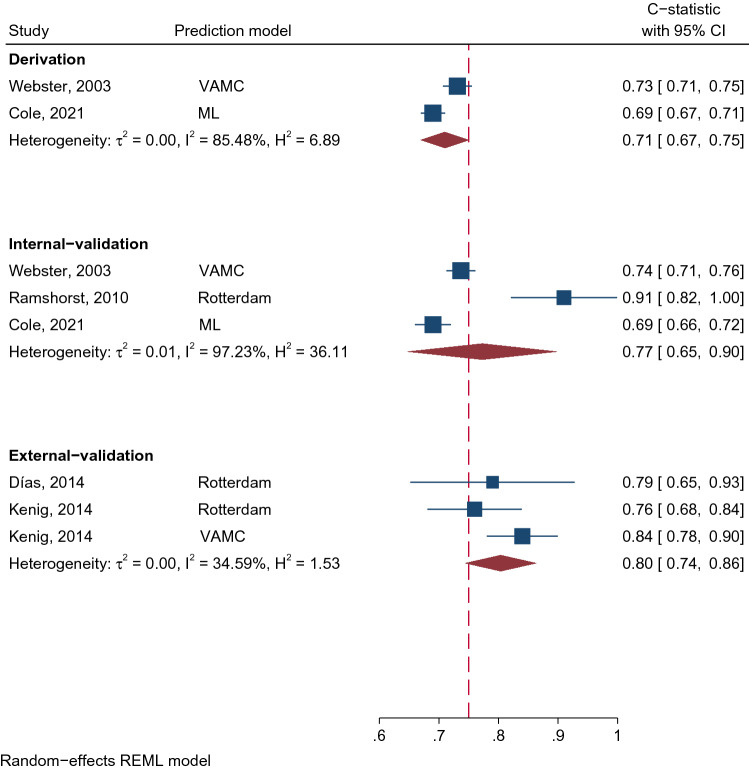
C-statistics of fascial dehiscence prediction models, where a higher C-statistic means a better discrimination performance. Dash line indicates the overall pooled C-statistic value. (VAMC Veteran Affairs Medical Centre, ML Machine Learning, REML restricted maximum likelihood)

The VAMC [17] model was constructed using logistic regression with backward stepwise elimination, and included 12 out of 22 initial risk factors, see Table 2. The model was internally validated using a split-data approach, and also externally validated by Kenig et al. [21]. Discrimination performance C-statistics (95% CI) in the derivation, internal-validation, and external-validation [17, 21] phases were 0.73 (0.71, 0.75), 0.74 (0.71, 0.76) [17], and 0.84 (0.78, 0.90) [21], respectively, see Fig. 2. Calibration performance was assessed using Hosmer–Lemeshow goodness-of-fit, which yielded p-values of 0.61, 0.82, and 0.46 in the derivation, internal-validation [17], and external-validation phase [21], respectively.

**Table 2:** Included predictive factors in each prediction model

Predictor variable	Study									
	Fascial dehiscence			Incisional hernia						
	Webster, 2003	Ramshorst, 2010	Cole, 2021	Veljkovic, 2010	Goodenough, 2015	Basta, 2016	Fischer, 2016	Lanni, 2016^a^	Tecce, 2017	Basta, 2019
*Pre-operative factor*										
Age		◉#	◉			◉#	◉#			
Sex		◉	◉							
Ethnicity							◉	◉		◉
BMI			◉	◉#	◉#	◉#	◉#	◉#	◉#	◉#
ASA status			◉							
2 + Elixhauser Comorbidity										◉
Chronic obstructive pulmonary disease	◉	◉	◉		◉					◉
Smoking			◉				◉		◉	◉
Coughing		◉								
Cerebrovascular accident	◉									
Ascites		◉							◉	
Jaundice		◉								
Anemia		◉							◉	
Hypertension			◉							
Cancer								◉		◉
Chemotherapy							◉			◉
Malnutrition						◉	◉			
Chronic liver disease							◉			
Alcohol abuse							◉			
Antiplatelet/anticoagulant										◉
Steroid			◉							
Prior hernia								◉		
History of abdominal surgery						◉				◉
*Intra-operative factor*										
Emergency operation	◉	◉	◉							◉
Open surgery					◉	◉		◉		◉
Midline incision									◉	
Hand-assisted laparoscopy					◉					
Organ of surgery		◉					◉			
Concurrent fistula/ostomy							◉			◉
Concurrent gastrointestinal procedure									◉	
Emergent vascular procedure										◉
Laparoscopic hysterectomy										◉
Small bowel obstruction										◉
Gynecologic pathology							◉		◉	
Acute inflammation							◉		◉	
Operative time	◉#		◉							
Suture length: Incision length				◉#						
Surgeon’s experience	◉									
Wound class	◉		◉							
*Post-operative factor*										
Reoperation	◉									
Time to stitch removal				◉#						
SSI	◉	◉	◉	◉						
Wound complication							◉			
Pneumonia	◉									
Failure to wean	◉									
Any complication	◉									
*Laboratory factor*										
Sodium			◉							
Creatinine			◉							
Hematocrit			◉							

*ASA*: American society of anesthesiologists, *BMI*: body mass index, *SSI*: Surgical site infection

^#^Used in categorized form

^a^Full-text not available – not all predictors reported

The Rotterdam model [18] was also constructed using logistic regression with backward elimination, and the final model included 10 risk factors (Table 2). The C-statistic from the derivation phase was not reported but it was 0.91 (0.81, 1.00) for the internal-validation (split-data approach), see Fig. 2. Hosmer–Lemeshow goodness-of-fit indicated that the model was well calibrated (*p*-value = 0.79). The Rotterdam model’s discrimination performance was lower in the external-validation studies, with C-statistics (95% CI) of 0.79 (0.65, 0.93) [20] and 0.76 (0.68, 0.84) [21] relative to 0.91 in the internal-validation phase. None of the studies provided calibration coefficients as an O/E ratio. The VAMC and Rotterdam risk score equations are provided in Online Resource Table S3.

The Virginia study group [28] recently developed a FD prediction model by applying machine learning (ML) approach using a decision-tree with extreme gradient boosting technique. Of 29 predictive factors, 15 were selected and kept in the ML model. Interestingly, this approach included laboratory data (i.e., serum sodium, creatinine, and hematocrit level) as potential predictive factors, see Table 2. Discrimination C-statistics (95% CI) were 0.69 (0.67, 0.71) and 0.69 (0.66, 0.72) in training and internal-validation sets, respectively [28] (Fig. 2). The model had good calibration but has yet to be externally validated.

C-statistics were pooled across studies stratified by study phases (see Fig. 2), which yielded pooled C-statistics (95% CI) of 0.71 (0.67, 0.75), 0.77 (0.65, 0.90), and 0.80 (0.74, 0.86) for derivation, internal-validation, and external-validation phases, respectively. This indicated that these prediction models performed better in the internal- and external-validation phases, although they were not significant. However, heterogeneity was very high with the corresponding degree of heterogeneity I^2^s of 85.5%, 97.2%, and 34.6%, respectively.

### IH prediction

Seven risk prediction models were developed for IH occurrence after general abdominal surgery [19, 22, 24, 27], bariatric surgery [23], colectomy [25], and hysterectomy [26]. These models were derived from the data of 428 to 30, 865 patients, followed up for more than 12 months, with maximum mean follow-up time of 57.9 months (Table 1).

**Fig. 3:**
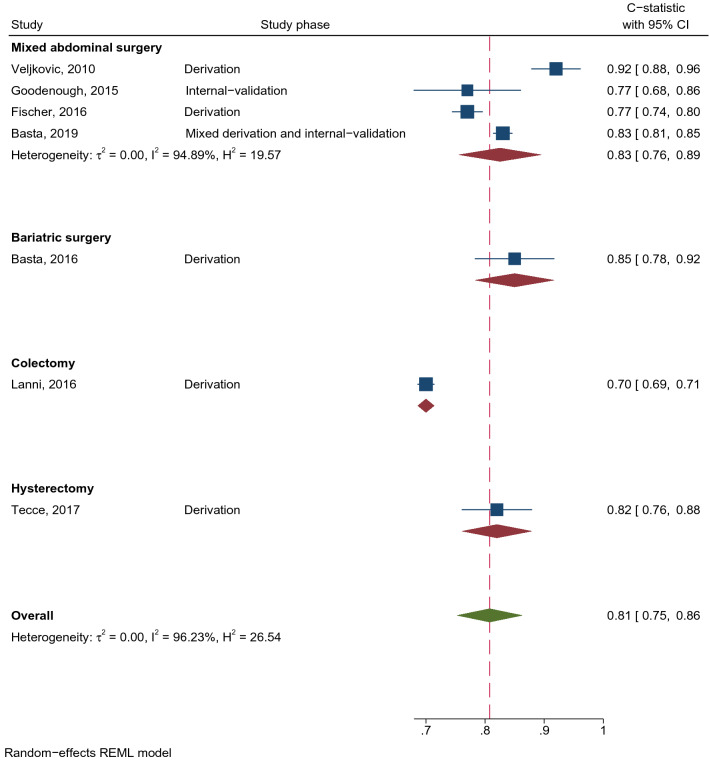
C-statistics of incisional hernia prediction models, where a higher C-statistic means a better discrimination performance. Dash line indicates the overall pooled C-statistic value. (REML restricted maximum likelihood)

Veljkovic et al. [19] developed an IH prediction model in midline laparotomy patients using logistic regression. The model was constructed from four predictors including BMI, suture length to incision length ratio, time to suture removal, and surgical site infection (SSI), see Table 2. Excellent discrimination performance was indicated from the C-statistic (95% CI) of 0.92 (0.88, 0.96), see Fig. 3. Calibration performance was reported as good but no statistic was reported.

HERNIAscore [22] is a well-known IH prediction model derived using Cox regression. It includes four predictive factors (i.e., BMI, chronic obstructive pulmonary disease (COPD), laparotomy, and hand-assisted laparoscopy) after model selection using backward elimination (see Table 2). No C-statistic was reported from the derivation phase but it was 0.77 (0.68, 0.86) from the split-sample internal-validation [22] (Fig. 3). The study did not state whether calibration performance was assessed.

Five IH prediction models [23–27] were developed by the same group from the University of Pennsylvania Health System. Four out of five models were derived from Cox regression with bootstrapping for model validation. C-statistics ranged from 0.70 to 0.85 [23–26] (Fig. 3). A recent model by this study group, named Penn hernia risk calculator [27], was deployed as a free mobile application. The model included 16 predictive factors, of which emergency laparotomy was weighted as the strongest risk factor (Table 2). C-statistic (95% CI) was 0.83 (0.81, 0.85) in the overall cohort (Fig. 3), or 0.84 and 0.82 in the derivation and split-sample internal-validation cohorts, respectively. Excellent calibration performance was claimed although no statistic was reported. This application allows users to estimate the risk of IH occurrence specific to different types of operation, including bariatric, colorectal, gastric, gynecological, hepatobiliary, transplant, vascular, and urological surgery. None of the IH prediction studies reported sensitivity, specificity, predictive values, and O/E ratio. IH risk score equations are shown in Online Resource Table S3.

C-statistics (95% CI) of the IH prediction models were pooled stratified by type of surgery (see Fig. 3) which yielded a pooled C- statistic of 0.81 (0.75, 0.86) for overall abdominal surgery with high heterogeneity *I*^2^ of 96.2%. In addition, a pooled C-statistic (95% CI) was 0.83 (0.76, 0.89) in mixed-abdominal surgery whereas there was only one each for Bariatric, colectomy, and hysterectomy.

## Discussion

Risk prediction models for FD or IH occurrence were systematically reviewed in this study. Three models were derived for FD considering a total of 26 risk factors, with discriminative performance (i.e., C-statistics) ranging from 0.69 to 0.73 for derivation, 0.69 to 0.74 for internal-validation, and 0.76 to 0.84 for external-validations. For IH prediction, a total of 32 risk factors were used in seven models, with discriminative C-statistics ranging from 0.70 to 0.92 for derivation, and 0.77 to 0.82 for internal-validation. Pooled C-statistics of the FD models were 0.77 and 0.80 in internal- and external-validation phases, and 0.81 for IH in derivation-internal-validation phase but these were highly heterogeneous leading to uncertainty, i.e., fair to excellent performance for both FD and IH models.

The Rotterdam model [18] and HERNIAscore [22] were derived based on relatively small cohorts (1, 452 and 428 patients, respectively), whereas newer models were developed from larger cohorts that utilized electronic medical records and a registered database [24, 27, 28]. As a general rule, more precision was observed from the large cohorts than small cohorts (Figs. 2 and 3). All FD and IH risk prediction models had high RoB according to the PROBAST criteria, especially within the analysis domain. Altogether, certainty of the evidence was rated as *very low* as per the GRADE approach [16].

Predictive factors commonly included in the FD models were emergency operation, COPD, and SSI [17, 18, 28]; the latter was consistently the strongest risk factor, with an odds ratio (OR) of 5.54 [17] to 6.43 [18]. For IH occurrence, BMI [19, 22–27] was the most commonly included factor, followed by surgical approach (i.e., laparotomy or laparoscopy) [22, 23, 25, 27], history of smoking [24, 26, 27], and ethnicity [24, 25, 27]. In the Penn hernia risk calculator, emergency operation was the most important risk factor, with an OR (95% CI) of 4.65 (3.90, 5.55) [27].

A prognostic prediction model could play a prominent role in determining whether to target additional intra-operative strategies and resources to reduce the risk of adverse outcomes in specific patient groups**.** There is existing evidence that procedures such as small-bite fascia closure [29] and prophylactic mesh placement [9, 11, 30] related to abdominal wall closure can reduce the risk of FD and IH occurrence following abdominal operations; however, mesh procedures require expertise, are time-consuming, and represent additional cost. Therefore, models based solely on factors available during the pre- or intra-operative phase could help target these additional resources, in contrast to prediction models [17–19, 28] dependent on postoperative predictive factors which would not be helpful.

Minimally invasive surgery is currently the preferred approach; this is supported by the fact that many models identified open surgery as an IH risk factor [22, 23, 25, 27]. However, the open procedure is still valuable in emergency situations, inevitably making emergency surgery a greater risk for FD [17, 18, 28] and IH [27]. This group of patients should benefit from prophylactic mesh placement. Although prophylactic mesh did not increase SSI risk in the recent clinical trial [31], most surgeons are still reluctant to use mesh in an emergency setting for fear of an SSI. If an emergency patient with a substantially high risk of FD and IH occurrence were identifiable using a risk prediction rule, it may guide the surgeon to determine which patients might benefit most from the use of prophylactic mesh despite the risk of SSI. Nevertheless, none of the prediction models were developed explicitly for emergency patients.

Other preoperative risk factors used in many models [22–27] are worth further consideration including obesity and smoking. Weight reduction and smoking cessation preoperatively should be encouraged to diminish the risk of IH. In addition, measures to reduce the risk of SSI should also reduce the risk of FD [17, 18, 28] including glycemic control [32], intra-operative normothermia [33], and antibiotic prophylaxis. Timing of antibiotic administration is crucial to ensure that the effective concentration of the antibiotic is achieved in tissues by the incision time [34]. Moreover, multiple doses of antibiotics should be delivered during the long procedure to maintain maximum protective effect [35]. While an individual’s risk estimation using prediction models requires complex calculation and might not be applied routinely, not adhering to basic principles, including small-bite closure, should be condemned.

Prediction models were mostly constructed using conventional statistical techniques such as logistic and Cox regression. Many risk factors were simultaneously considered in the equations to increase predictive performance; however, there are a number of caveats in using these models. Too many risk factors relative to the number of events and/or a total sample size can cause model over-fitting, with a consequent loss of generalizability. A common rule of thumb is that one needs 10–30 events per risk factor to reduce overfitting with logistic regression. In addition, nonlinear relationships and interaction between risk predictors should also be considered in model development to improve model performance; it is well known that conventional statistical models may be limited in dealing with these issues, particularly in the presence of high-dimensional interactions. Unlike conventional statistical methods, ML methods can easily address these issues in addition to multi-collinearity between risk factors. Nevertheless, the black box nature of ML methods makes clinical interpretation more difficult. In our review, only one ML-based model was identified.

Overall, the predictive performance for most models was still too low to be adopted into clinical practice. In general, a predictive model should have an AUC of at least 0.8 or preferably 0.85 to be sufficiently precise and accurate to justify its use in clinical practice, with subsequent validation in an independent population. When discrimination performance (i.e., C-statistic) was considered, less precise models should be used with caution. The model with a higher C-statistic and precision, e.g., Penn hernia risk calculator [27], may be a good model for external validation.

This review has some strengths. Eligible studies were systematically identified and selected. The RoB was assessed using the most appropriate and validated [36] tool (i.e., PROBAST) designed explicitly for prediction models. However, limitations cannot be avoided. Most publications included in this review were judged as having a high risk of bias. In addition, high heterogeneity was observed from the pooling of FD and IH prediction model performance (i.e., C-statistics), thus uncertainty of this performance was present. Due to the limited number of studies/models available, neither subgroup analysis by type of incision (open versus laparoscopy) nor type of abdominal surgery (bariatric surgery, colectomy, and hysterectomy) could be done. We did not initially propose to assess risk of bias using the PROBAST in the PROSPERO; applying it after critical appraisal of included studies might lead to bias the results.

In conclusion, several models have been developed for FD and IH risk predictions but most of them had high risk of bias. Their performances are highly heterogeneous, which vary from fair to excellent for both models. Further studies are required to externally validate these models before applying them in a routine clinical practice. In addition, these models may need to be updated with additional important risk factors and tailored to specific patient populations, such as emergency abdominal operations.

## Supplementary Information

Below is the link to the electronic supplementary material.

Supplementary file1 (PDF 280 KB)

Supplementary file2 (DOCX 32 KB)

Supplementary file3 (XLSX 13 KB)

## Data Availability

All data are available from previously published articles and Online Appendix 2.

## Abbreviations

BMI: Body mass index
CI: Confidence interval
COPD: Chronic obstructive pulmonary disease
C-statistic: Concordance statistic
FD: Fascial dehiscence
GRADE: Grade of recommendation, assessment, development, and evaluation
IH: Incisional hernia
ML: Machine learning
O/E: Observed/expected
OR: Odds ratio
RoB: Risk of bias
ROC: Receiver operating characteristic
SSI: Surgical site infection
VAMC: Veteran affairs medical centre
