# Supplementary material for: Fascial Dehiscence and Incisional Hernia Prediction Models: A Systematic Review and Meta-analysis
Source: World J Surg. 2022 Sep 14;46(12):2984–95. doi: 10.1007/s00268-022-06715-6 (PMC9636101; doi:10.1007/s00268-022-06715-6)
Supplement: Supplementary file 1 — Supplementary file1 (PDF 280 KB) [file 268_2022_6715_MOESM1_ESM.pdf]

World Journal of Surgery

Electronic Supplementary Material

Fascial dehiscence and incisional hernia prediction models: A systematic review and meta-analysis

Amarit Tansawet, Pawin Numthavaj, Thawin Techapongsatorn, Suphakarn Techapongsatorn, John Attia, Gareth McKay, Ammarin Thakkinstian

Corresponding author
Pawin Numthavaj, M.D., Ph.D.
Department of Clinical Epidemiology and Biostatistics, Faculty of Medicine, Ramathibodi Hospital, Mahidol University
Rama VI Road, Ratchathewi, Bangkok, Thailand 10400
Tel: +6622011762
Fax: +6622011284
Email: pawin.num@mahidol.ac.th

Table legends

Table S1. Search terms
Table S2. Risk of bias of the included studies
Table S3. Risk score equations

Table S1. Search terms

Database	Search term
Scopus	TITLE-ABS-KEY (((((((((incision*) OR (postoperative)) OR (ventral)) OR (scar)) AND (hernia*)) OR (((dehiscence) OR (eviscerat*)) OR ("burst abdomen"))) AND (((((predict*) OR (prognos*)) OR (risk)) OR (stratif*)) AND (((model) OR (score)) OR (index)))) AND (((((((((("receiver operating characteristic") OR (roc)) OR (auc)) OR (auroc)) OR ("concordance statistic")) OR ("c-statistic")) OR (accuracy)) OR (sensitivity)) OR (specificity)) OR (((validat*) OR (deriv*)) OR (develop*)))) AND (LIMIT-TO (DOCTYPE , "ar") OR LIMIT-TO (DOCTYPE , "re"))
Medline (PubMed)	(((((((incision*) OR (postoperative)) OR (ventral)) OR (scar)) AND (hernia*)) OR (((Dehiscence) OR (eviscerat*)) OR ("burst abdomen"))) AND (((((Predict*) OR (prognos*)) OR (risk)) OR (stratif*)) AND (((model) OR (score)) OR (index)))) AND (((((((((("receiver operating characteristic") OR (ROC)) OR (AUC)) OR (AUROC)) OR ("concordance statistic")) OR ("c-statistic")) OR (accuracy)) OR (sensitivity)) OR (specificity)) OR (((validat*) OR (deriv*)) OR (develop*)))
Web of Science	(((((((incision*) OR (postoperative)) OR (ventral)) OR (scar)) AND (hernia*)) OR (((Dehiscence) OR (eviscerat*)) OR ("burst abdomen"))) AND (((((Predict*) OR (prognos*)) OR (risk)) OR (stratif*)) AND (((model) OR (score)) OR (index)))) AND (((((((((("receiver operating characteristic") OR (ROC)) OR (AUC)) OR (AUROC)) OR ("concordance statistic")) OR ("c-statistic")) OR (accuracy)) OR (sensitivity)) OR (specificity)) OR (((validat*) OR (deriv*)) OR (develop*)))

Table S2. Risk of bias of the included studies

PROBAST		Fascial dehiscence					Incisional hernia						
Domain	Signal question	Webster 2003	Ramshorst 2010	Cole 2021	Días 2014	Kenig 2014	Veljkovic 2010	Goodenough 2015	Basta 2016	Fischer 2016	Lanni 2016*	Tecce 2017	Basta 2019
Participants	Were appropriate data sources used?	Y	N	Y	N	N	Y	Y	N	N		N	N
	Were all inclusions and exclusions of participants appropriate?	Y	N	Y	Y	Y	Y	Y	Y	Y		Y	Y
	Summary domain 1	+	-	+	-	-	+	+	-	-		-	-
Predictors	Were predictors defined and assessed in a similar way for all participants?	PY	NI	PY	PY	PY	PY	PY	PY	PY		PY	PY
	Were predictor assessments made without knowledge of outcome data?	PY	PY	PY	PY	PY	PY	Y	Y	Y		Y	Y
	Are all predictors available at the time the model is intended to be used?	Y	N	Y	Y	Y	Y	Y	Y	Y		Y	Y
	Summary domain 2	+	-	+	+	+	+	+	+	+		+	+
Outcome	Was the outcome determined appropriately?	Y	PY	Y	PY	Y	NI	Y	PY	PY		PY	PY
	Was a pre-specified or standard outcome definition used?	Y	PY	Y	PY	Y	NI	Y	Y	Y		Y	Y
	Were predictors excluded from the outcome definition?	Y	Y	Y	Y	Y	Y	Y	Y	Y		Y	Y
	Was the outcome defined and determined in a similar way for all participants?	Y	PY	Y	NI	Y	PY	N	NI	NI		NI	NI
	Was the outcome determined without knowledge of predictor information?	PY	PY	PY	PY	PY	NI	NI	Y	Y		Y	Y
	Was the time interval between predictor assessment and outcome determination appropriate?	Y	Y	Y	NI	Y	N	PY	Y	Y		Y	Y
	Summary domain 3	+	+	+	?	+	-	-	?	?		?	?
Analysis	Were there a reasonable number of participants with the outcome?	Y	Y	Y	N	N	N	N	N	Y		N	Y
	Were continuous and categorical predictors handled appropriately?	Y	N	Y	Y	Y	N	N	PN	PN		PN	PN
	Were all enrolled participants included in the analysis?	NI	Y	NI	NI	NI	N	NI	Y	Y		Y	Y
	Were participants with missing data handled appropriately?	PN	PN	PN	NI	N	N	PN	NI	NI		NI	N
	Was selection of predictors based on univariable analysis avoided?	N	N	Y			N	N	N	N		N	N
	Were complexities in the data accounted for appropriately?	Y	Y	Y	Y	Y	Y	Y	Y	Y		Y	Y
	Were relevant model performance measures evaluated appropriately?	N	N	Y	N	N	N	N	N	N		N	N
	Were model overfitting and optimism in model performance accounted for?	PN	N	Y			N	N	Y	Y		Y	PN
	Do predictors and their assigned weights in the final model correspond to the results from multivariable analysis?	Y	Y	PY			N	Y	Y	Y		Y	Y
	Summary domain 4	-	-	-	-	-	-	-	-	-		-	-
**Overall**		high	high	high	high	high	high	high	high	high		high	high

* No available full-text

Table S3. Risk score equations

Study	Equation
Webster, 2003	Score = 4*(CVA/Stroke no deficit) + 4*(History COPD) + 4 *(Current pneumonia) + 6*(Emergency procedure) + 2*(Operative time > 2.5 h) + 3*(Post graduate year 4 Resident) – 3*(Clean wound classification) + 5*(Superficial wound infection) + 17*(Deep wound infection) + 6*(Failure to wean) + 7*(One or more complications) – 11*(Return to OR)
Ramshorst, 2010	Score = 0.4*(Age 40-49) + 0.9*(Age 50-59) + 0.9*(Age 60-69) + 1.1*(Age >70) + 0.7*(Male) + 0.7*(Chronic pulmonary disease) + 1.5*(Ascites) + 0.5*(Jaundice) + 0.7*(Anemia) + 0.6*(Emergency surgery) + 0.7*(Gallbladder/Bile duct surgery) + 1.5*(Esophageal surgery) + 1.4*(Gastroduodenal surgery) + 0.9*(Small bowel surgery) + 1.4*(Large bowel surgery) + 1.3*(Vascular surgery) + 1.4*(Coughing) + 1.9*(Wound infection)
Veljkovic, 2010	Score = 32*(Suture incision ratio < 4.2) + 30*(SSI CDC 2 or 3) + 9*(Time to suture removal > 16 d) + 2*(BMI > 24 kg/m^2)
Goodenough, 2015	Score = 4*(Laparotomy) + 3*(HAL) + 1*(COPD) + 1*(BMI ≥ 25 kg/m^2)
Basta, 2016	Score = 5*(Open bariatric approach) + 2*(Malnutrition) + 2*(History of abdominal surgery) + 2*(BMI ≥ 60 kg/m^2) + 1*(Age 45-65)
Fischer, 2016	Score = 3*(Hispanic or Native American) + 2*(White) – 1*(Asian) + 3*(Concurrent ostomy/fistula takedown) + 1*(Concurrent ostomy creation) + 2*(Recent chemotherapy) + 2*(Obesity) – 1*(Normal weight) + 2*(Bariatric procedure) + 2*(Proctectomy) + 1*(Partial colectomy) + 1*(Small bowel resection) + 2*(History of alcohol abuse) + 2*(History of smoking) + 2*(History of liver disease) + 1*(Acute inflammatory process) + 1*(History of surgical wound complication) + 1*(Malnutrition) + 1*(Age > 45 yr) – 1*(Benign gynecologic mass)
Tecce, 2017	Score = 2*(Vertical incision) + 1*(Ascites) + 1*(Gynecologic malignancy) + 1*(BMI > 30 kg/m^2) + 1*(Acute inflammatory process) + 1*(Anemia) + 1*(Smoking history) + 1*(Concurrent GI procedure)
Basta, 2019	Score = 4*(Emergency laparotomy) + 2*(History of abdominal surgery) + 2*(Emergent vascular procedure) + 2*(Caucasian) + 1*(Indication: SBO) + 1*(Smoker) + 1*(2+ Elixhauser comorbidities) + 1*(Open approach) + 1*(BMI > 30 kg/m^2) – 2*(BMI 18-25 kg/m^2) – 4*(BMI < 18 kg/m^2) + 1*(Chronic liver disease) + 1*(History of cancer) + 1*(History of chemotherapy/XRT) + 1*(Concurrent fistula/ostomy procedure) + 1*(ASA/Anticoagulant use) + 1*(Chronic pulmonary disease) – 2*(Laparoscopic hysterectomy)
